# Dynamic Network Connectivity: from monkeys to humans

**DOI:** 10.3389/fnhum.2024.1353043

**Published:** 2024-02-07

**Authors:** Amy F. T. Arnsten, Min Wang, Mark D’Esposito

**Affiliations:** ^1^Department of Neuroscience, Yale School of Medicine, New Haven, CT, United States; ^2^Department of Psychology, Helen Wills Neuroscience Institute, University of California, Berkeley, Berkeley, CA, United States

**Keywords:** prefrontal cortex, catecholamines, working memory, fMRI, acetylcholine, glutamate, NMDA

## Abstract

Human brain imaging research using functional MRI (fMRI) has uncovered flexible variations in the functional connectivity between brain regions. While some of this variability likely arises from the pattern of information flow through circuits, it may also be influenced by rapid changes in effective synaptic strength at the molecular level, a phenomenon called Dynamic Network Connectivity (DNC) discovered in non-human primate circuits. These neuromodulatory molecular mechanisms are found in layer III of the macaque dorsolateral prefrontal cortex (dlPFC), the site of the microcircuits shown by Goldman-Rakic to be critical for working memory. This research has shown that the neuromodulators acetylcholine, norepinephrine, and dopamine can rapidly change the strength of synaptic connections in layer III dlPFC by (1) modifying the depolarization state of the post-synaptic density needed for NMDA receptor neurotransmission and (2) altering the open state of nearby potassium channels to rapidly weaken or strengthen synaptic efficacy and the strength of persistent neuronal firing. Many of these actions involve increased cAMP-calcium signaling in dendritic spines, where varying levels can coordinate the arousal state with the cognitive state. The current review examines the hypothesis that some of the dynamic changes in correlative strength between cortical regions observed in human fMRI studies may arise from these molecular underpinnings, as has been seen when pharmacological agents or genetic alterations alter the functional connectivity of the dlPFC consistent with the macaque physiology. These DNC mechanisms provide essential flexibility but may also confer vulnerability to malfunction when dysregulated in cognitive disorders.

## Introduction

“*The great topmost sheet of the mass, that where hardly a light had twinkled or moved, becomes now a sparkling field of rhythmic flashing points with trains of traveling sparks hurrying hither and thither. The brain is waking and with it the mind is returning. It is as if the Milky Way entered upon some cosmic dance. Swiftly the head mass becomes an enchanted loom where millions of flashing shuttles weave a dissolving pattern, always a meaningful pattern though never an abiding one; a shifting harmony of subpatterns.*”

(Sherrington, 1940).

**FIGURE 1:**
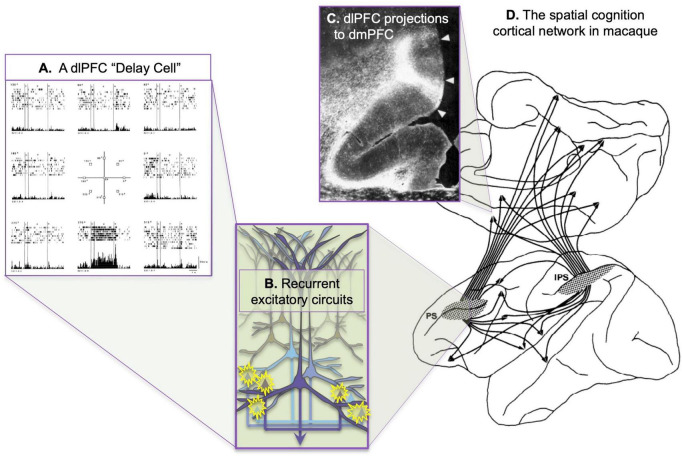
The cortical networks of visual spatial cognition in the macaque as discovered by Goldman-Rakic and Schwartz (1982). **(A)** An example of a dlPFC “Delay cell,” which represents the lower spatial location in working memory. This neuron fires across the delay period for every trial in which the monkey must remember the location of 270 degrees, but not for other spatial locations. From Funahashi et al. (1989). **(B)** Extensive local recurrent excitation in deep layer III of the dlPFC is likely a major generator of persistent firing during working memory, with longer-range recurrent excitatory connections to areas such as the ipsilateral parietal association cortex and the contralateral dlPFC. Based on the extensive, local horizontal connections shown in deep layer III (Kritzer and Goldman-Rakic, 1995). **(C)** An example of dlPFC projections to the dmPFC, showing the columnar nature of the termination. **(D)** The spatial cognition network, showing the shared connectivity patterns of the dlPFC surrounding the principal sulcus (PS) and the parietal association cortex in the intraparietal sulcus (IPS). **(B,C)** Adapted from Selemon and Goldman-Rakic (1988).

Goldman-Rakic (1987) transformed the field of cognitive neuroscience by helping to uncover the underlying organization of the “Enchanted Loom”- the warp of parallel sensory inputs continuing forward into the prefrontal cortex (PFC), the weft of local recurrent excitatory circuits capable of sustaining neuronal firing without any sensory stimulation, generating the “possibility that concepts and plans can govern behavior,” an evolutionary advance in higher cognition. She was able to illuminate the networks of spatial cognition and the role of the dorsolateral prefrontal cortex (dlPFC) in generating representations of spatial information in working memory, providing the first cellular basis for a higher cognitive operation (Goldman-Rakic, 1995). She built on the work of Fuster (1973) before her to discover “Delay cells” in the dorsolateral PFC (dlPFC) that could represent visual space in the absence of sensory stimulation, the foundation of abstract thought (Figure 1A). And she revealed the cellular bases for this fundamental capability by studying the intrinsic and extrinsic connections of the dlPFC, discovering microcircuits with extensive recurrent excitation within deep layer III (Figure 1B), interconnected with extensive cortico-cortical connections (Figures 1C–D).

**FIGURE 2:**
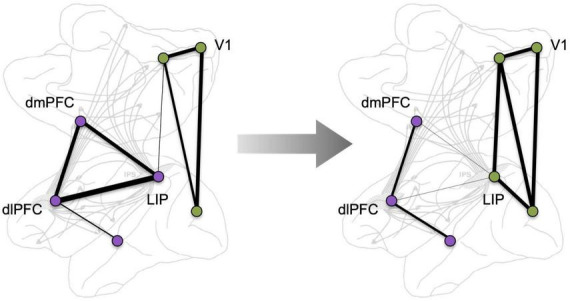
A schematic depiction of the dynamic nature of cortical connectivity, where network correlated activity changes, e.g., in response to a cognitive challenge. Background adapted from Selemon and Goldman-Rakic (1988).

The roles of higher cortical networks in cognition are studied at different levels of analysis among neuroscientists (e.g., molecular, cellular, systems), dictated by the tools available for study in different species [e.g., single-unit recording in macaques vs. functional MRI (fMRI) in humans]. With the great expansion of the neurosciences, these arenas have all too often lost contact with each other, making it difficult to link the complexities of molecular signaling mechanisms and cellular dynamics obtained from macaque studies to findings at the regional and system level obtained from human studies. Moreover, the enormity of the number of human brain imaging studies in the past 25 years is intimidating to any neuroscientist who attempts to link studies with different levels of analysis. This contrasts with the time of Goldman-Rakic’s research, when human brain imaging was just emerging and was closely wed to the basic science, with each area readily benefiting from this cross-pollination (Curtis and D’Esposito, 2003). Today, these fields have their own seemingly independent inertial forces. But, as so often happens in evolution, these separate fields seem to have discovered a similar phenomenon: the very dynamic nature of cortical connectivity, e.g., as schematically illustrated in Figure 2. These changes can occur in a matter of moments, too quickly to involve structural changes, and they are key for flexible cognition that can be coordinated with instantaneous environmental demands and with fluctuations in arousal state. Data from macaques described more fully below, suggest that Sherrington’s (1940) “dissolving pattern” is greatly influenced by the molecular environment, e.g., where the “traveling sparks” of axon potentials may continue their course from one neuron to the next only if in the presence of the appropriate, very local, molecular milieu: e.g., dependent on sufficient extracellular acetylcholine to permit NMDA neurotransmission, and sufficient intracellular regulation of cAMP-calcium signaling to maintain closure of the nearby potassium (K^+^) channels that dissolve the transfer of information (Arnsten et al., 2021). This review will examine whether some of these dynamic molecular events in the macaque dlPFC can be related to dynamic changes in functional connectivity seen in human brain imaging studies.

Before we proceed, an important cautionary note regarding the term “connectivity.” It should be emphasized that functional *connectivity* measured by fMRI is actually functional *correlativity*, as even sophisticated scientists can confuse correlations between regional activities with real anatomical connections. Similarly, the physiological studies in monkeys infer a change in connectivity as an underlying contribution to changes in neuronal firing, given Delay cells requirement for recurrent excitation to maintain firing across the delay period. This review will try to be explicit in describing the correlativity of human fMRI measures vs. the anatomical measures of connections in the macaque as we try to relate data across these different, but tightly related, fields, as well as reminders of the limitations of single unit recordings, which probe only a single node in what is likely a very complex network.

## Primate cortical neuroanatomy, a brief review

**FIGURE 3:**
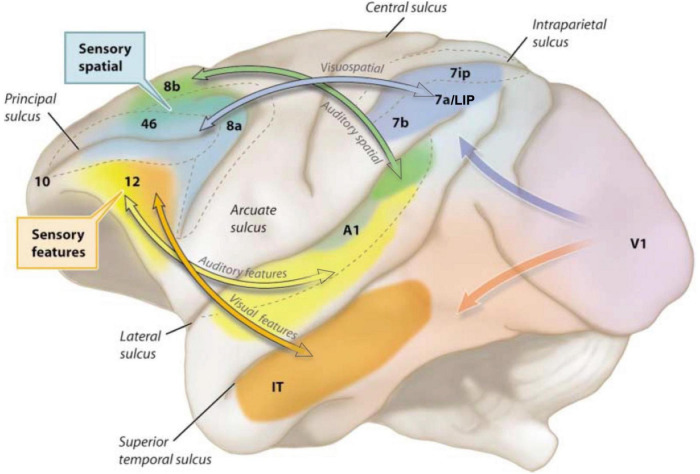
The modular sensory streams in macaque cortex continue into the PFC, with a dorsal zone receiving inputs about visual and auditory space, and ventral zones receiving inputs about visual and auditory features. Adapted from Arnsten (2003); original image created by S. Mark Williams based on Goldman-Rakic (1987).

Goldman-Rakic revealed the architecture of long-range cortical connections in macaque, e.g., in her work with Selemon identifying a spatial cognition network interconnecting the dlPFC with the parietal association cortices and their many shared targets (Figure 1; Selemon and Goldman-Rakic, 1988). Goldman-Rakic (1987) showed that the parallel organization of sensory processing established in posterior cortices extended into the PFC, whereby visual and auditory *spatial* information targeted more dorsal lateral zones, while visual and auditory *feature* information projected into more ventral lateral areas (Figure 3). There were also extensive reciprocal projections back to parietal and temporal cortices to provide “top-down” control of sensory processing (Selemon and Goldman-Rakic, 1988; Rossi et al., 2007). It is important to note that the simple laminar scheme of feedforward vs. feedback projections generally breaks down at these higher levels (Barbas et al., 2018), where projections often show a columnar pattern traversing across layers (Goldman-Rakic and Schwartz, 1982; Selemon and Goldman-Rakic, 1988), e.g., as seen in Figure 1C. These complexities are often not appreciated in the literature, where an over-simplified, canonical circuit can falsely represent the real details of cortico-cortical communication.

**FIGURE 4:**
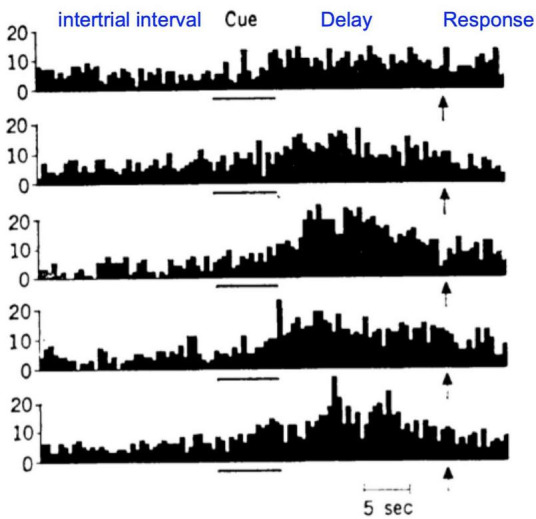
An example of a dlPFC Delay cell recorded by Fuster and Alexander (1971) during a manual version of a visuospatial working memory task, with long intertrial intervals and long delays. Each row shows the neuron firing on a different trial. As this version of the task does not require visual fixation, longer delays are feasible. This was the first study to report increased firing during the delay period in dlPFC neurons, and set the stage for Goldman-Rakic’s studies showing the spatial tuning based on retinal coordinates (Figure 1A).

The anatomical data showing parallel sensory streams into the PFC were consistent with Goldman-Rakic’s physiological data, where “Delay cells” representing visual space (Figure 1A) were found more dorsally, and those representing visual features more ventrally (Wilson et al., 1993). Human studies using fMRI found a similar dorsal to ventral pattern (Ungerleider et al., 1998). Importantly, Delay cells are able to continue firing across the delay period in a working memory task even though the cue is no longer present. For example, in a visuospatial working memory task, Delay cells fire across the delay period for the memory of its preferred spatial location but not for other locations, i.e., they are spatially tuned (Figure 1A; Funahashi et al., 1989). Tasks typically utilize relatively short delays, e.g., 5 s, before the next trial begins and the contents of working memory must be updated with a new spatial location. The original studies by Fuster (1973) using a manual spatial working memory task (and thus not limited by the need to fixate the eyes) found delay cells that could continue firing for 18 s (e.g., Figure 4); lesion data suggest that longer delays require interactions with the hippocampus (Zola-Morgan and Squire, 1985). Delay cell persistent firing is accompanied by a more global signature in the local field potential, with bursts in the gamma frequency range as a defining signature (Bastos et al., 2018).

Goldman-Rakic explored the local connectivity within the dlPFC that might provide these circuits with their remarkable ability to continue firing without sensory stimulation. She showed that deep layer III of the dlPFC was the site of microcircuits with extensive horizontal connections (Kritzer and Goldman-Rakic, 1995), forming recurrent excitatory networks to maintain persistent firing across the delay period in a working memory task (Figure 1B), with lateral inhibition from GABAergic interneurons to refine spatial tuning (Goldman-Rakic, 1995). There were also horizontal connections in superficial layer V, although not as extensive, while deep layer V showed the canonical local labeling pattern confined to a single column (Kritzer and Goldman-Rakic, 1995). Goldman-Rakic’s hypothesis regarding recurrent excitation was confirmed by González-Burgos et al. (2000) using *in vitro* recordings from dlPFC, and it is likely that the expansion of this anatomical feature is key to the rise of cognitive abilities in primates. It is noteworthy that persistent firing can be seen in other nodes of the spatial cognition network, e.g., in the lateral intraparietal (LIP) cortex, but the persistent firing in LIP is vastly reduced when the dlPFC is temporarily inactivated, while the converse has only subtle effects (Chafee and Goldman-Rakic, 2000). Thus, the dlPFC is a key generator of the persistent firing needed for working memory. This is an important, general point: physiological recordings and fMRI can show reflected as well as generative neural activity, whereas causal manipulations are required to truly denote the true contribution of a node in a network.

It is critical to note that Delay cells are mostly active, i.e., functionally connected, during working memory compared to at rest (Fuster and Alexander, 1971; Funahashi et al., 1989). This is best illustrated in the work of Fuster (1973), where the manual version of the spatial working memory task had long intertrial intervals, thus allowing comparison of “spontaneous firing” to firing patterns once a trial was initiated, e.g., as shown in Figure 4. As Fuster wrote in his landmark paper (Fuster and Alexander, 1971), “Almost all the units investigated (57 in MD, 110 in prefrontal cortex) showed rather irregular patterns of spontaneous firing while the animal was at rest during intertrial periods…, the majority of units.increased their spike activity to levels higher than those prevalent in intertrial periods.with some reaching discharge levels more than tenfold higher than the spontaneous discharge level.” As we will see below, pharmacological agents that affect Delay cell firing often have large effects on firing during the delay epoch of the task, but little effect on spontaneous firing or firing during the fixation period.

## What is Dynamic Network Connectivity?

Dynamic Network Connectivity refers to the rapid changes in network strength between neurons in primate dlPFC due to the rapid and powerful actions of neuromodulators near the synapse which can gate neurotransmission and the flow of information through dendritic spines (for detailed reviews, see Arnsten et al., 2010, 2012). Thus far, this research has been performed studying the cortex of the rhesus macaque, and has only been able to examine changes at a single site, mostly within the dlPFC. This work is finding that the levels of norepinephrine, dopamine, and acetylcholine released in the dlPFC determine whether Delay cell circuits are able to connect and generate the firing needed for working memory or are disconnected, as occurs with uncontrollable stress exposure (serotonin’s actions are still poorly understood in primate dlPFC but likely contribute greatly as well). Thus, these neuromodulators have an enormous effect on the connectivity of dlPFC microcircuits, which is critical for cognition. As described below, these mechanisms determine whether there is effective N-methyl-D-aspartate receptor (NMDAR) neurotransmission and effective transfer of the depolarized signal from the spine to the parent dendrite, altering the routing of information within a cortical network, e.g., by opening potassium channels on spines to gate out incoming information. Thus, these neuromodulators can silence or excite the actions of dlPFC microcircuits, including gating out network inputs to refine the contents of working memory (Vijayraghavan et al., 2007).

**FIGURE 5:**
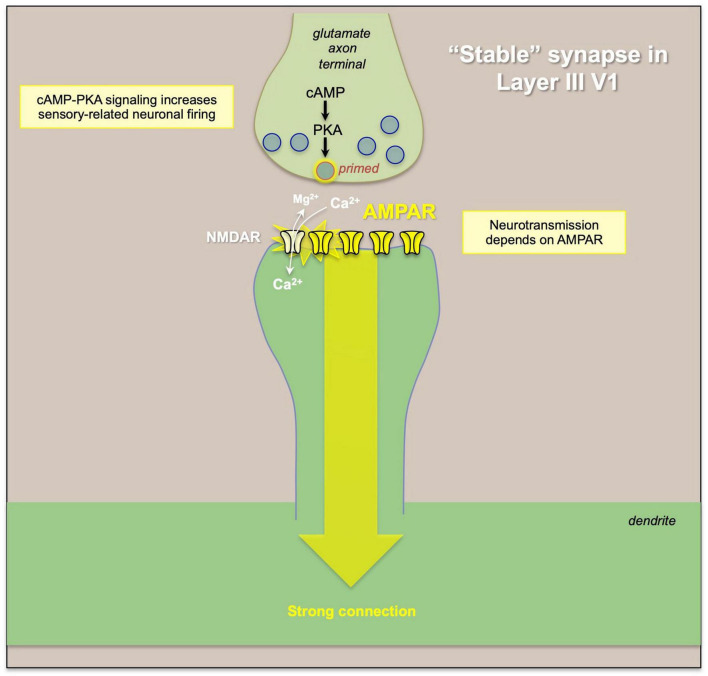
An example of “typical” glutamate signaling at a “stable” synapse, where glutamate release from the presynaptic terminal activates AMPAR, which depolarize the synaptic membrane to eject the magnesium (Mg^2+^) from NMDA receptor pore, permitting NMDAR neurotransmission. Thus, the strength of the connection and of neural firing is primarily determined by the activity of the presynaptic neuron and the amount of glutamate release.

These powerful modulatory actions in the dlPFC contrast with generally subtler effects on neurons in primary visual cortex (area 17, i.e., V1), where neurons evaluate visual stimuli even under conditions of anesthesia and encode sensory events despite differing arousal states (Figure 5). For example, dopamine has little or no influence on V1 neuronal firing in macaques (Zaldivar et al., 2014), but has enormous effects on dlPFC neuronal firing, as described below. It is noteworthy that cholinergic mechanisms do modulate V1 neuronal responses (e.g., Disney et al., 2012; Herrero and Thiele, 2021), but these actions are more subtle in V1 than their marked, permissive actions in dlPFC as described in the following section.

**FIGURE 6:**
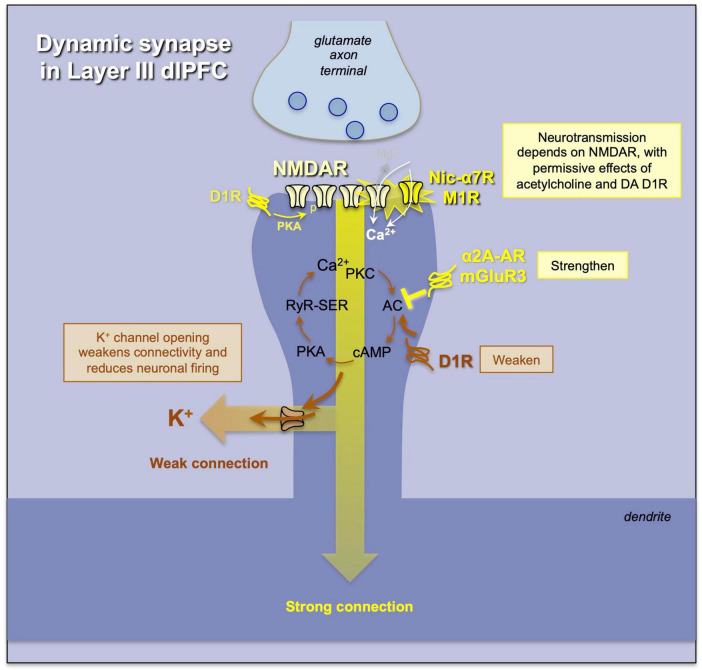
An example of a “dynamic” synapse in layer III of macaque dlPFC, where the strength of the connection and the degree of neuronal firing are determined by (1) the amount of acetylcholine release permitting NMDAR neurotransmission, and (2) the regulation of cAMP-calcium-K^+^ channel signaling, where K^+^ channel opening weakens connectivity and reduces firing, while inhibition of cAMP-calcium-K^+^ channel signaling strengthens network connectivity and enhances neuronal firing needed for higher cognition. Neuromodulators interface with these mechanisms to determine the strength of dlPFC function.

In classical glutamate synapses, such as those in primate V1 (Figure 5), α-amino-3-hydroxy-5-methyl-4-isoxazolepropionic acid receptors (AMPAR) are permissive for NMDAR neurotransmission, depolarizing the synaptic membrane to relieve the magnesium (Mg^2+^) block of the NMDAR pore and allowing NMDAR neurotransmission. In contrast, AMPAR have relatively subtle effects on dlPFC Delay cell firing (Wang et al., 2013), and these critical permissive actions are instead carried out by acetylcholine stimulation of nicotinic α7-receptors (Yang et al., 2013) and muscarinic M1 receptors (Galvin et al., 2020), which are localized within the glutamate post-synaptic membrane (Figure 6). Thus, blockade of nicotinic α7-receptors or muscarinic M1 receptors prevents NMDA from exciting the cell, and markedly diminishes Delay cell firing (Yang et al., 2013; Galvin et al., 2020). dlPFC Delay cells are wholly dependent on NMDAR neurotransmission, including synaptic NMDAR with GluN2B subunits that flux high levels of calcium (Wang et al., 2013), and both nicotinic α7-receptors (Yang et al., 2013) and muscarinic M1 receptors (Galvin et al., 2020) also increase calcium levels. Dopamine D1 receptors (D1R) are also found within the glutamatergic post-synaptic density (Arnsten et al., 2015), and dopamine stimulation of D1R is also needed for Delay cell firing (Williams and Goldman-Rakic, 1995; Wang et al., 2019), possibly by PKA phosphorylation of the NMDAR that allows calcium flux through the NMDAR pore, and/or to maintain the NMDAR within the membrane (Arnsten et al., 2015; Figure 6). The high levels of calcium near the synaptic membrane may be needed to maintain membrane depolarization necessary for NMDAR actions (Arnsten et al., 2021). Thus, these modulatory actions are required for layer III dlPFC neurotransmission, and the absence of acetylcholine release during deep sleep may explain why we are unconscious during this state, as without cholinergic-NMDAR neurotransmission, dlPFC circuits would be “off-line” (Wang et al., 2013). It is not known if this reliance on NMDAR, acetylcholine and D1R for neurotransmission is apparent in other association cortices, as this in depth analysis has only been performed in the dlPFC. However, there is an increasing gradient of *GRIN2B* (NMDAR-GluN2B), *CALB1* (calbindin), and *DRD1* (dopamine D1R) expression across the cortical hierarchy (Burt et al., 2018; Froudist-Walsh et al., 2021), consistent with their greater role in dlPFC, and suggesting that other higher cortical areas at the top of the hierarchy may also have molecular mechanisms that render them more reliant on neuromodulatory state (Arnsten et al., 2021).

Layer III dlPFC pyramidal cells also have unusual, powerful neuromodulation that allows for rapid changes in network connectivity. As schematized in Figure 6, the dendritic spines of layer III pyramidal cells also express potassium channels that are opened by high levels of feedforward cAMP-PKA-calcium signaling (Wang et al., 2007; Arnsten et al., 2010, 2012). When these channels open, they effectively “gate out” synaptic inputs, reducing the recurrent excitation needed for Delay cell firing. Many neuromodulators appear to interact with this mechanism to regulate levels of neuronal firing. For instance, there are dopamine D1Rs on the spine membrane near these channels, at a distance from the synapse, and they likely activate cAMP-PKA-calcium signaling to refine network inputs to a cell, narrowing its tuning properties (Vijayraghavan et al., 2007; Paspalas et al., 2013; Gamo et al., 2015). For example, KCNQ2 potassium channels are opened by PKA signaling (Galvin et al., 2020), and HCN channels are opened by cAMP, and appear to couple with Slack K^+^ channels to ultimately increase K^+^ efflux (Wu et al., 2023). In this way, moderate levels of dopamine D1R stimulation can sculpt away non-preferred inputs to create more precise mental representations (Vijayraghavan et al., 2007), while high levels of dopamine D1R and norepinephrine α1-AR activation, as occurs with uncontrollable stress exposure, can markedly reduce Delay cell firing, likely by cAMP-calcium opening of nearby K^+^ channels (Birnbaum et al., 2004; Gamo et al., 2015; Datta et al., 2019). Conversely, α2A-adrenoceptors and metabotropic glutamate receptor-3 (mGluR3) inhibit cAMP-PKA-calcium signaling and close K^+^ channels, strengthening synaptic connectivity needed for recurrent excitation and thus enhancing memory-related firing (Wang et al., 2007). This likely contributes to the therapeutic effects of α2A-adrenoceptor agonists such as guanfacine in treating clinical disorders thought to be due to PFC dysfunction (Arnsten, 2020; Arnsten et al., 2023).

**FIGURE 7:**
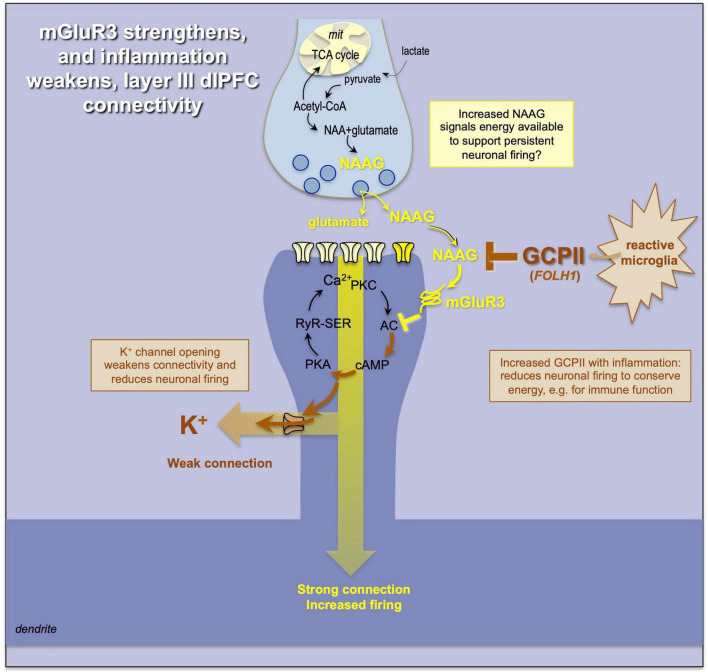
mGluR3 signaling has a particularly powerful post-synaptic role in primate dlPFC, inhibiting cAMP-K^+^ channel opening and enhancing cognition. mGluR3 are stimulated by NAAG as well as glutamate; NAAG may act as an index of energy availability and foster persistent firing. In contrast, the release of GCPII under inflammatory conditions would destroy NAAG and reduce neuronal firing, reserving energy for the immune system.

The effects of mGluR3 are of particular interest, as these mechanisms have changed and expanded their role over PFC evolution (Figure 7). In contrast with rodent PFC, where mGluR3 are predominately presynaptic (Woo et al., 2022), in primate dlPFC mGluR3 have a large post-synaptic role where they strengthen synaptic connectivity by inhibiting cAMP-K^+^ channel signaling, enhancing delay firing and improving cognitive function (Jin et al., 2018). mGluR3s are not only stimulated by glutamate but by *N*-acetyl-aspartyl-glutamate (NAAG), which is co-released with glutamate and is selective for mGluR3 amongst the glutamate receptors (Vornov et al., 2016; Neale and Olszewski, 2019). NAAG is also released from astrocytes (Vornov et al., 2016), and may be an index of energy availability, as it is synthesized from acetyl co-A, e.g., an index of Krebs cycle metabolism. We have speculated that NAAG stimulation of mGluR3 may be a way that astrocytes communicate that there is energy availability to support persistent neuronal firing (Yang et al., 2022).

The loss of mGluR3 protective actions also appears to be an important component of the cognitive deficits that occur with inflammation (Figure 7). Under inflammatory conditions, e.g., COVID infection, microglia synthesize and release the enzyme glutamate carboxypeptidase II (GCPII) (Zhang et al., 2016; Arteaga Cabeza et al., 2021), which destroys NAAG, and causes a marked loss of dlPFC neuronal firing (Yang et al., 2022). GCPII is encoded by the *FOLH1* gene, and a gain-of-function mutation in *FOLH1* is associated with impaired cognition in human subjects (Zink et al., 2020), consistent with its important role in higher cortical function in primates. Recent data from aged macaques additionally suggest that GCPII inhibition may help to reduce tau pathology, consistent with inflammation contributing to the neuropathology of sporadic Alzheimer’s disease (Bathla et al., 2023). Cognitive disorders such as schizophrenia are also linked to alterations in the *GRM3* gene that encodes mGluR3 (Egan et al., 2004), highlighting the importance of this mechanism to human cognition.

**FIGURE 8:**
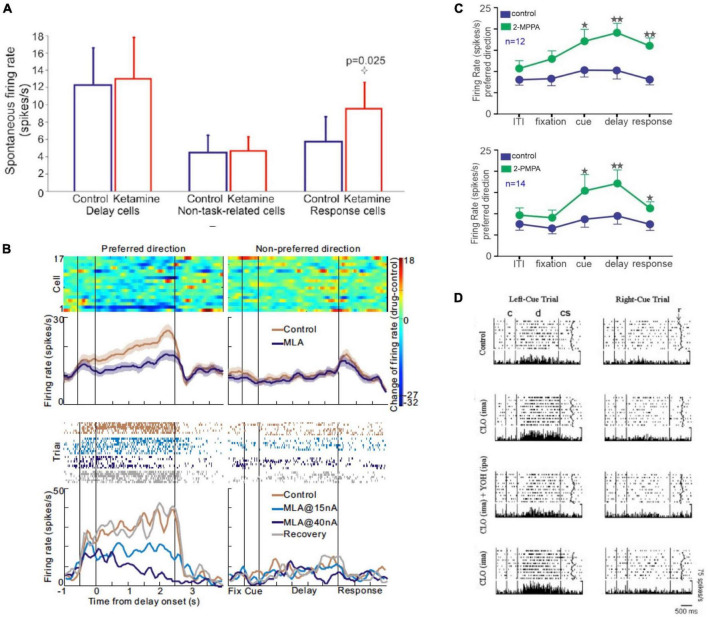
Examples where pharmacological manipulation altered macaque dlPFC neuronal firing during the delay period, but not during the intertrial interval and/or fixation period. **(A)** The effects of the NMDAR antagonist, ketamine, on spontaneous firing of macaque dlPFC neurons. Ketamine, which reduced Delay cell firing during working memory, had no effect on spontaneous Delay cell firing during rest. In contrast, Response feedback cells showed increased firing during both task performance and at rest. From Wang et al. (2013). **(B)** The nicotinic α7R antagonist, MLA, reduced Delay cell firing during the delay period, but had no effect during the fixation period. The top figure shows the average of 17 dlPFC Delay cells; the bottom is an example neuron. From Yang et al. (2013). **(C)** The GCPII inhibitors 2-MPPA and 2-PMPA markedly increase Delay cell firing during the delay period, as well as during the cue and response periods, but do not significantly alter firing during the intertrial interval or the fixation period. From Yang et al. (2022). **(D)** Systemic administration of the α2-AR agonist, clonidine, significantly increased firing during the delay period but not during fixation or baseline. The effects of clonidine were reduced by local, iontophoretic delivery of the α2-AR antagonist, yohimbine, onto the recorded neurons. From Li et al. (1999).

As mentioned above, most of these modulatory effects on dlPFC Delay cell firing are only seen under conditions of cognitive engagement, and do not significantly alter neuronal firing during the intertrial interval or the fixation period when working memory operations are not engaged. For example, the NMDAR antagonist, ketamine, has no effect on Delay cell firing under conditions of rest, but markedly reduces Delay cell firing when the monkey is performing a working memory task (Wang et al., 2013; Figures 8A). Similarly, iontophoresis of the nicotinic α7R antagonist, MLA, markedly reduced neuronal firing during the delay period, but not during fixation (Figure 8B; Yang et al., 2013). Other examples are seen with the GCPII inhibitors, 2-MPPA or 2-PMPA, which increase firing during the cue, delay and response epochs, but not during the intertrial interval or the fixation period (Figure 8C; Yang et al., 2022). This specificity was also reported for systemic administration of the α2-AR agonist, clonidine, which enhanced firing during the delay period but did not alter baseline firing (Figure 8D; Li et al., 1999). Together, these findings highlight that the modulatory effect of neuromodulators on cortical activity patterns may not be evident during rest or other conditions where their cognitive operations are not engaged. Note that these specific alterations during cognitive engagement are not true for all cell types, e.g., post-saccadic “feedback cells” in dlPFC showed increased firing to ketamine even under non-task conditions (Wang et al., 2013). Thus, this is a heterogeneous feature of cortical neurons, which would integrate in the fMRI BOLD signal. To compare physiological studies of macaques with human brain imaging studies, it is important to consider these findings at the cellular level. The following sections will highlight examples where similar changes in neuronal firing/functional connectivity are seen in studies of macaque and human cortex.

## Relating to dynamic connectivity in humans

Dynamic Functional Connectivity, a term coined by researchers performing human brain functional imaging, has qualities in common with the Dynamic Network Connectivity studied in macaques. However, the macaque and human research approach the question of dynamic changes from differing levels, where each has an advantage and a disadvantage. Although the human studies are unable to perform molecular manipulations at the cellular level, the human fMRI work has the significant advantage of having the capacity to investigate connectivity between distant brain regions with exceptional spatial and temporal precision, as it enables comprehensive whole-brain measurements. In contrast, obtaining whole-brain data in macaques poses a challenge, although there is a growing body of research employing depth electrodes spanning multiple brain regions and dural grid placement across extensive cortical regions, (e.g., Corrigan et al., 2022). fMRI experiments can test hypotheses about interactions between brain regions by focusing on covariances of activation levels between regions (Buckner et al., 2013). These covariances reflect “functional connectivity,” a concept originally developed regarding temporal interactions among individual neurons (Gerstein et al., 1978).

Functional connectivity methods used in fMRI studies are of two types, determined by whether the method assesses connectivity in a model-free or model-based fashion. Model-free methods, referred to as “functional” connectivity methods, measure the temporal covariance in activity between brain areas without *a priori* notions about which brain areas are relevant or how they should interact (e.g., Corrigan et al., 2022). Examples include correlation and its frequency-based analog coherence. Model-free methods, the most implemented method in fMRI studies, can also be categorized as “static” or “time-varying.” Time-varying functional connectivity is often referred to in the imaging literature as “dynamic” functional connectivity, which is not to be confused with how we use this term in this paper (Lurie et al., 2020). Static functional connectivity analyses examine correlations between the BOLD responses averaged across an entire experimental session of data collection, assuming that connectivity between brain regions does not vary with time. In contrast, time-varying connectivity analyses examine fluctuations in functional connectivity over the course of an experimental session of data collection. Model-based methods, referred to as “effective” connectivity methods, begin with hypotheses about the interactions between different brain regions and attempt to support/refute them by evaluating the presence/absence of specific activity covariance patterns. Examples include structural equation modeling and dynamic causal modeling (Penny et al., 2004).

Mathematical tools based on graph theory have emerged as another method to assess brain connectivity, allowing for quantification of large-scale network properties of the brain as well as identifying the role of individual brain regions within these large-scale networks. For example, a graph-theory metric called “modularity” quantifies the extent to which individual brain subnetworks, or network modules, are segregated from other modules in the whole brain network, where networks with high modularity have many connections within modules and sparser connections between modules (Sporns and Betzel, 2016). Graph theory methods can also measure other metrics of network function, such as shifts in network memberships, where a node that initially correlates with one network may transition to correlating with another (Hutchison et al., 2013), e.g., as schematized in Figure 2. These dynamic network variations have been linked to changes in cognitive functioning, such as learning (e.g., Sun et al., 2007; Bassett et al., 2015).

For model-free, model-based, and graph theoretic approaches, fMRI data can be collected while the subject is performing or not performing a task, commonly referred to as the “resting state.” However, it should be noted that the “resting state” is not the same as the “fixation” period used in cellular studies to measure baseline neural activity. It is important to consider the exact cognitive state of the subject when comparing findings from human imaging and macaque physiological studies. This will ensure a more meaningful comparison between the two and help to draw accurate conclusions.

## Dynamic Network Connectivity and Dynamic Functional Connectivity: translating from monkeys to humans

Comparing data from various levels of analysis, such as moving from single-unit recordings in monkeys to human fMRI data, poses a challenge, yet one that is surmountable. Undoubtedly, the groundbreaking research conducted by Goldman-Rakic laid the foundation for countless human fMRI studies of cognition. Recent advances in our understanding of the molecular and cellular underpinnings of Dynamic Network Connectivity, as elucidated above, allow one to form hypotheses that can be tested with human fMRI data that measure Dynamic Functional Connectivity.

A major caveat with single cell recordings in monkeys is that we do not know the identitie(s) of the input(s) exciting the neuron we are recording from. The anatomy suggests that these likely include local recurrent excitatory inputs from neighboring pyramidal cells, as well as longer range connections e.g., with the contralateral dlPFC, with LIP and/or MD thalamus. No one yet has been able to record and/or stimulate one cortical area while recording from an interconnected dlPFC neuron during iontophoresis, a feat that would require heroic efforts and a great deal of luck. However, as Delay cells require persistent synaptic excitation to maintain firing across the delay period, these experiments can give us a general sense of how neurochemical state can profoundly alter the ability of a neuron to functionally contribute to circuit activity, which may manifest as fluctuations in functional connectivity measured with fMRI.

Performing cognitive tasks during fMRI scans in conjunction with pharmacological challenges can reveal significantly different insights compared to using each method separately. When these two approaches are combined, referred to as pharmacological fMRI, it is possible to uncover neurochemical mechanisms underlying human cognition. Pharmacological fMRI involves comparing the effects of drugs versus placebo on the modulation of brain activity induced by cognitive tasks. These drug-task interactions indicate a modulation of the underlying anatomical and chemical systems within the brain rather than reflecting only unspecific cerebrovascular effects. Drugs that modulate catecholamines, acetylcholine, and NMDAR are available in humans and can be safely administered in single doses during fMRI scanning. However, the FDA has not approved drugs for modulation of all neurochemical systems. For example, a selective dopamine D1R agonist is not yet available. Also, since these drugs are administered orally or intravenously, they cannot target specific brain regions but rather will act on the entire brain. Nevertheless, pharmacological MRI in humans allows for testing predictions regarding Dynamic Network Connectivity derived from empirical work in macaques regarding neurochemical influences on Delay cell firing. In the following sections, we present selected examples of findings from human pharmacological fMRI studies that align with predictions derived from macaque studies.

### Glutamate

Glutamate neurotransmission is much of the “starlight” on Sherrington’s (1940) enchanted loom. As described above, Delay cells in dlPFC largely depend on NMDAR neurotransmission, with permissive effects of acetylcholine (Figure 6; Wang et al., 2013). In contrast, layer V Response-feedback cells in macaque dlPFC depend on both NMDAR and AMPAR (Wang et al., 2013). These two different cell types respond differently to NMDAR blockade with systemic ketamine: Delay cells show REDUCED firing, *but only under conditions of cognitive engagement and not during rest*, while Response cells show disinhibited firing during both a cognitive task or at rest (Wang et al., 2013; Figure 8). The disinhibition of Response cells likely involves the blockade of NMDAR on GABA interneurons, irrespective of the cognitive state (Goldman and Rosvold, 1970; Rotaru et al., 2012).

These findings predict that the administration of the NMDA receptor antagonist, ketamine, to humans would decrease the functional connectivity of dlPFC during the execution of a working memory task, a change that would not be observed when subjects are not actively involved in the task. Consistent with this prediction, in a human fMRI study (Driesen et al., 2013), ketamine administration reduced dlPFC connectivity during the performance of a spatial working memory task, and the magnitude of these functional connectivity changes were related to performance. The effect of ketamine on functional connectivity when the subject was not performing the working memory task was not investigated. In another human fMRI study of healthy humans during a resting state (Mueller et al., 2018), ketamine administration *increased* functional connectivity between the dlPFC and the anterior cingulate. Thus, cognitive state has a large effect on how the dlPFC responds to ketamine in both monkeys and humans.

Another example of parallel effects of glutamate signaling in macaques and humans is the findings regarding the role of metabotropic glutamate receptors on primate cognition. As described above, the glutamate receptor mGluR3 is stimulated by NAAG, which GCPII catabolizes under conditions of inflammation. In macaque, GCPII markedly reduces Delay cell firing due to loss of mGluR3 regulation of cAMP-K^+^ channel signaling (Figure 7; Jin et al., 2018; Yang et al., 2022). Humans with a gain-of-function mutation in *FOLH1*, (the gene that encodes for GCPII) have reduced levels of NAAG in dlPFC, as measured by MRI spectroscopy, and impaired visual memory and IQ scores that correlated with reduced NAAG expression (Zink et al., 2020). Although functional connectivity of the dlPFC was not measured in this study, the inefficient cortical activation during working memory in humans and the detrimental effects of GCPII on dlPFC Delay cell firing in macaques shows interesting parallels across species.

### Catecholamines

Methylphenidate and atomoxetine increase both norepinephrine and dopamine in PFC (the norepinephrine actions of MPH are often forgotten due to studying its actions in the striatum where there is little norepinephrine) (Bymaster et al., 2002; Berridge et al., 2006; Spencer et al., 2012). Atomoxetine is of special interest as it does not increase dopamine in the striatum. Low doses of atomoxetine increase Delay cell firing in macaques by enhancing endogenous norepinephrine α2A-AR and dopamine D1R stimulation (Gamo et al., 2010). These findings in macaques predict that administering these drugs to humans would increase the functional connectivity of dlPFC during the execution of a working memory task. Consistent with this prediction, a human fMRI study (Hernaus et al., 2017) found that atomoxetine administration increased functional connectivity between the dlPFC and the insula during the most demanding cognitive condition of a working memory task, and this correlated with behavioral performance. In another human fMRI study, atomoxetine (van den Brink et al., 2016) enhanced functional connectivity of the inferior frontal gyrus with the striatum during a response inhibition task, the PFC region most linked to improved stopping in macaques and humans (Zhukovsky et al., 2022). Finally, in a human fMRI study of resting state data investigating large-scale networks (Shine et al., 2018), the administration of atomoxetine shifted network configuration that was maximal in the lateral frontal cortex, amygdala and visual cortex.

### Norepinephrine

Both the α2-AR agonist clonidine (Li et al., 1999), and the more selective α2A-AR agonist guanfacine (Wang et al., 2007) enhance the firing of dlPFC Delay cells in macaques. Thus, these findings predict that administering these drugs to humans would increase the functional connectivity of dlPFC during the execution of a working memory task. Consistent with this prediction, in a human positron emission tomography study, clonidine administration had differing effects on functional connectivity based on cognitive state. During rest, clonidine decreased the functional strength of connections both from the frontal cortex to the thalamus and in pathways to and from visual cortex, while during an attentional task, functional connectivity increased, e.g., from parietal cortex to thalamus and frontal cortex (Coull et al., 1999). Similarly, in a human fMRI study, modulated task-related functional connectivity of the amygdala with PFC that was associated with the emotional biasing of cognitive control processes (Schulz et al., 2014).

### Dopamine

Dopamine mechanisms are more problematic to test in humans, as there are no selective D1R agonists approved for human use at the time of this writing. The data in macaques show an inverted-U dose-response with dopamine D1R actions on dlPFC Delay cells (Vijayraghavan et al., 2007, 2016, 2017; Arnsten et al., 2015; Wang et al., 2019). Most pharmacological fMRI studies in humans have used the mixed D1R/D2R agonist bromocriptine and have seen a similar inverted-U dose-response in behavior, activity, and functional connectivity (for a review, see Cools and D’Esposito, 2011). For example, after dopaminergic augmentation with bromocriptine, frontal–striatal connectivity in individuals with low working memory capacity individuals increased, corresponding with behavioral improvement, whereas decreases in connectivity in individuals with high working memory capacity individuals were associated with poorer behavioral performance (Wallace et al., 2011). Similarly, during the performance of a delayed match-to-sample task with face stimuli, bromocriptine altered distracter-resistance, impairing performance after face relative to scene distraction. Across individuals, this drug effect correlated negatively with drug effects on delay period signal in the PFC and functional connectivity between the PFC and the fusiform face area (Bloemendaal et al., 2015).

### Aging

Studies in macaques have revealed that Delay cells in dlPFC show reduced persistent firing with advancing age due to excessive cAMP-calcium-K^+^ channel signaling (Wang et al., 2011), which would weaken the recurrent excitation needed to generate and sustain neuronal firing across the delay period of a working memory task, including long-range recurrence with the parietal association cortex (Chafee and Goldman-Rakic, 2000; Suzuki and Gottlieb, 2013). This finding predicts a decline in functional connectivity between dlPFC and parietal cortex during normal aging in humans. Although various human fMRI studies have demonstrated age-related changes in functional network connectivity (for a review, see Sala-Llonch et al., 2015), one study directly addressed this hypothesis. In an fMRI study of healthy younger (20–32 years) and older (60–75 years) during the performance of a working memory task (Heinzel et al., 2017), weaker functional connectivity was found between the dlPFC and the parietal association cortex in older subjects with impaired working memory (Heinzel et al., 2017). Using dynamic causal modeling of the fMRI data to infer the direction of these interactions, it was also found that younger adults exhibited increased connectivity from dlPFC to parietal cortex during increased working memory load, which was not observed in older adults. These findings align with the hypothesis that a decline in dlPFC function with aging reduces feedback or top-down control of other brain regions (Gazzaley et al., 2005). This study underscores the strong concurrence with cellular-level discoveries in macaque monkeys as well as how human data can extend the model of Dynamic Network Connectivity originally derived from macaque data.

## Summary

In closing, Dynamic Network Connectivity and Dynamic Functional Connectivity appear to have much in common, and may be reflections of some of the same actions at the molecular level. The functioning of the PFC is particularly dependent on neurochemical state, where changes in neuromodulators determine whether synaptic inputs are effective or actively weakened. These molecular mechanisms may contribute to the dynamic nature of how PFC circuits contribute to wider networks. This bridge between the human and non-human primate research continues the extraordinary legacy of Goldman-Rakic, who first revealed the power of dopamine actions in dlPFC, and revealed the network basis for spatial cognition in primates.

## Author contributions

AA: Conceptualization, Funding acquisition, Writing – original draft, Writing – review & editing. MW: Writing – review & editing. MD’E: Conceptualization, Writing – review & editing.
